# Microvascular Alteration in COVID-19 Documented by Nailfold Capillaroscopy

**DOI:** 10.3390/diagnostics13111905

**Published:** 2023-05-29

**Authors:** Lucrezia Mondini, Paola Confalonieri, Riccardo Pozzan, Luca Ruggero, Liliana Trotta, Selene Lerda, Michael Hughes, Mattia Bellan, Marco Confalonieri, Barbara Ruaro, Francesco Salton, Stefano Tavano

**Affiliations:** 1 Pulmonology Unit, Department of Medical Surgical and Health Sciences, University Hospital of Cattinara, University of Trieste, 34149 Trieste, Italy; 2 Graduate School, University of Milan, 20149 Milano, Italy; 3 Division of Musculoskeletal and Dermatological Sciences, Faculty of Biology, Medicine and Health, The University of Manchester & Salford Royal NHS Foundation Trust, Manchester M6 8HD, UK; 4 Department of Translational Medicine, Università del Piemonte Orientale (UPO), 28100 Novara, Italy; 5 Center for Autoimmune and Allergic Disease (CAAD), Università del Piemonte Orientale (UPO), 28100 Novara, Italy; 6 Azienda Ospedaliero–Universitaria, Maggiore della Carità, 28100 Novara, Italy

**Keywords:** COVID-19, nailfold capillaroscopy, microcirculation

## Abstract

COVID-19 is a multisystemic disease that mainly affects and causes dysregulation of the endothelium, causing systemic manifestations. A nailfold video capillaroscopy is a safe, easy, and noninvasive method to evaluate microcirculation alteration. In this review, we analyzed the literature available to date regarding the object of nailfold video capillaroscopy (NVC) use in patients with a SARS-CoV-2 infection, both in the acute phase and after discharge. The scientific evidence pointed out the main alterations in capillary circulation shown by NVC, so reviewing the findings of each article allowed us to define and analyze the future prospects and needs for possibly including NVC within the management of patients with COVID-19, both during and after the acute phase.

## 1. Introduction

SARS-CoV-2 is a virus belonging to the Coronavirus family, which is responsible for a global pandemic that started 11 March 2020 [1]. The first cases of SARS-CoV-2 infection were recorded in Wuhan, China, and the disease quickly spread worldwide, causing various spectrums of clinical manifestations: from severe bilateral interstitial pneumonia requiring respiratory support and intensive care to mostly asymptomatic forms [2]. The scientific community then quickly focused on identifying the characteristics of COVID-19 disease and the most appropriate treatments, producing an enormous literature. Over time, the consequences of SARS-CoV-2 infection have manifested in some subgroups of patients as post-COVID-19 signs and symptoms, which is recognized as Long COVID syndrome [3]. The most severe forms of SARS-CoV-2 interstitial pneumonia, associated with acute respiratory distress syndrome (ARDS) and the need to manage patients in intensive care, are characterized by an aberrant inflammatory response, called a cytokine storm [4]. Understanding these mechanisms has made therapeutic protocols possible that are aimed at controlling the inflammatory pattern and predicting disease progression [5,6]. COVID-19 is characterized not only by pulmonary involvement but also by systemic involvement, including renal injury, myocardial damage, and pro-thrombotic evaluation. Based on this assumption, several studies highlighted the involvement of the endothelium in the development of the signs and symptoms of this disease, emphasizing that endothelial damage and microvascular dysfunction are central to its pathogenesis and the multiorgan damage it causes [7,8]. Analyzing the pathogenesis of SARS-CoV-2 infection showed how the prothrombotic status and hyperactivation of the immune system lead to the formation of immunothrombi, which is characterized by aggregates of neutrophils, platelets, and fibrin [9,10]. The presence of immunothrombi was confirmed in autopsy studies on patients with severe SARS-CoV-2 pneumonia, which were performed by Nicolai et al., that found neutrophil extracellular traps, together with aggregates of platelets and fibrin in the lung, kidneys, and heart of patients who died of COVID-19. Moreover, the analyzed groups presented with neutrophil-platelet aggregates and a neutrophil and platelet activation pattern in their blood, which is related to disease severity [10]. Platelet activation appears to be secondary to their interaction with anti-SARS-CoV-2 antibodies and to the elevated production of cytokines and molecules that are released due to extensive cellular damage, such as Adenosine Diphosphate and Adenosine Triphosphate, which act as thrombotic agonists [9,11].

**Figure 1:**
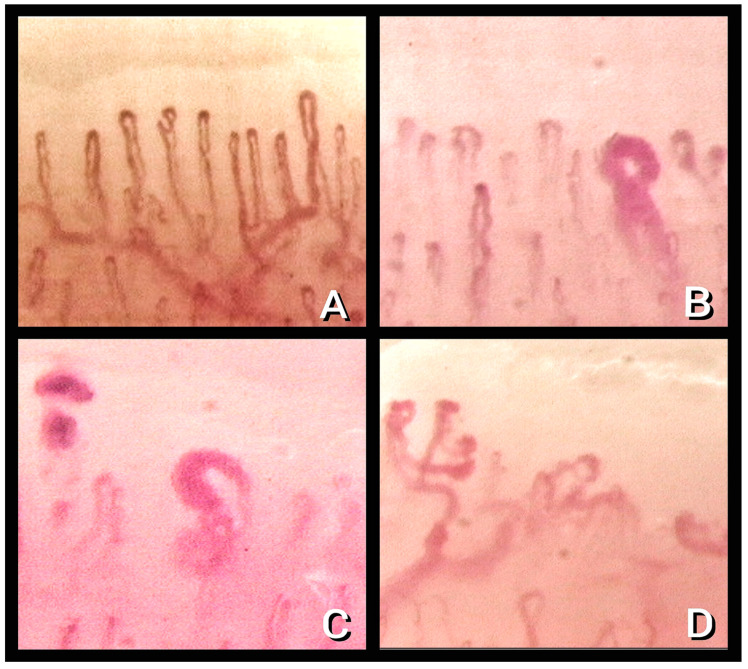
Nailfold video capillaroscopy images (×200) in healthy subjects (**A**) and “early” (**B**), “active” (**C**), and “late” (**D**) patterns of scleroderma microangiopathy (Operators: S. T., L. R., B.R. and L. M.).

The study of microcirculation alterations by nailfold video capillaroscopy (NVC) was, therefore, proposed by several authors. Figure 1 shows representative pictures of capillaries shown by NVC.

NVC, which has been used since the 1990s and was validated by the 2013 American College of Rheumatology (ACR)/Europe and League Against Rheumatism (EULAR) classification criteria for systemic sclerosis (SSc), represents the best method for analyzing microvascular abnormalities in rheumatologic diseases by reproducing amplified in vivo images of skin microcirculation [12,13,14]. Today, it is part of the normal clinical routine, as a useful tool in the diagnosis of connective tissue diseases to identify the scleroderma-like pattern, and is easily performed and noninvasive [15,16,17]. Since NVC appears to be a simple method for analyzing microcirculatory changes, its use has also been extended to non-rheumatic diseases with known endothelial involvement, such as diabetes, systemic hypertension, glaucoma, and sickle cell disease. Notably, the application of this method was also studied on pulmonary diseases, including interstitial lung disease, chronic obstructive pulmonary disease, and pulmonary hypertension, showing reduced capillary density and neoangiogenic phenomena [18,19,20]. With the advent of SARS-CoV-2 disease and emerging evidence of endothelial involvement, several studies recruited patients at different stages of COVID-19 to analyze microcirculation changes by NVC or focus on the sublingual microcirculation examined with a sidestream dark field camera [18,19].

The purpose of this review is to summarize the various works in the literature on this subject to understand the different stages of microcirculation alterations visible by NVC, starting from acute infection, according to different degrees of severity, to Long COVID forms. Building on this premise, the aspects that still need to be analyzed and explored and the inclusion of this method in clinical practice for the management of patients with SARS-CoV-2 infection are highlighted [20,21,22,23,24,25,26,27].

## 2. NVC Findings in COVID-19

The first study that examined microcirculation changes in patients at different stages of SARS-CoV-2 infection was conducted in Italy in 2021 by Natalello et al. Eighty-two patients with COVID-19, which was confirmed through blood tests (SARS-CoV-2 serology) and/or nasopharyngeal swab positivity and suggestive chest images, were recruited. In 28 patients, NVC was performed during hospitalization, while 54 patients were examined after discharge and then after the acute phase. Of the patients analyzed during hospitalization, 60.7% presented with respiratory failure that required oxygen therapy (via nasal cannula, via high-flow mechanical ventilation, or noninvasive), but none of them required admission to an intensive care unit [21]. Among the patients analyzed after discharge, 55.6% received oxygen therapy and 9.3% required ICU admission and mechanical ventilation. First, this study detected visible microcirculatory changes in SARS-CoV-2 infection by NVC, which were classified as nonspecific in 64.4% (*n* = 53) of cases. Second, based on the previous literature, some microvascular alterations previously described in patients undergoing NVC were distinguished, as listed in Table 1. The microcirculatory alterations in features 1–7 were examined through the semiquantitative scoring system proposed by Sulli et al., which was previously applied to microcirculatory alterations in patients with SSc: 0 = no alterations, 1 = less than 33% capillary alterations/reductions, 2 = 33–66% capillary alterations/reductions, and 3 = more than 66% capillary alterations/reductions, per linear millimeter [22]. A summary score was assigned for features 1–12: 0 = absent, 1 = present at least once in one finger, 2 = present at least once in 2–4 fingers, and 3 = present at least once in 5–8 fingers. All acutely ill patients examined during hospitalization showed pre-capillary edema (100%), sludge flow (78.6%), hemosiderin deposition (71.4%), and microvascular alteration (50%), none of which involved more than 33% of the analyzed capillaries. In the subgroups of discharged patients, NVC found dilated capillaries (85.2%), meandering capillaries (81.4%), pericapillary edema (70.4%), and low capillary density (63.0%) in more than two fingers. This study confirmed the hypothesis that the microcirculation changes observed by NVC were different depending on the stage of the disease. In addition, hemosiderin deposits supported the hypothesis that SARS-CoV-2 infection is characterized by diffuse micro thrombosis, which is especially present in the acute stages of the disease. This study represents the first examination of microcirculation by NVC in COVID-19 patients and took place in 2020, so the pharmacological protocols were refined as we went along. Therefore, evaluating the influence of various drug protocols on microcirculation changes is difficult to interpret [23]. This study laid the foundation for the use of NVC in COVID-19, demonstrating the utility of analyzing larger groups of patients, with different spectra of SARS-CoV-2 manifestation and possible alterations before and after COVID-19. Contextually, the correlation of capillaroscopic alterations with laboratory parameters could be useful in analyzing disease progression and predicting the development of complications, particularly related to the pro-thrombotic set up.

The first study introducing healthy control group was performed by Karahan et al., which compared the NVC findings in 32 patients in intensive care units with COVID-19-associated lung involvement; 29 patients without documented thromboembolism represented the healthy control. Eight capillaroscopy patterns were considered, as listed in Table 2. 

The most represented capillary pattern in the COVID-19 group was serpentine (56.2%), and the median capillary diameter was higher in the COVID-19 group than in the control (77.78 ± 3.63 μm vs. 71.67 ± 2.19 μm). In addition, there were significantly more enlarged capillaries, giant capillaries, avascular areas, microaneurysms, and microhemorrhages in the COVID-19 group. The only statistically significant difference between the two groups was the capillary density, which was lower in the COVID-19 group (6.41 ± 1.21/1 mm vs. 8.55 ± 1.12/1 mm in the control group). Furthermore, in this study it was shown that the capillary density was lower in patients who died of SARS-CoV-2 infection and was negatively correlated with D-dimer values. This study examined a population with more severe infection than the patient cohort analyzed by Natalello et al. However, a small number of patients who were not re-evaluated after hospital discharge were considered. Therefore, this analysis represents a single point in time in a small population; re-evaluation and the involvement of a larger number of subjects could provide more information [29].

Agabiti Rosei et al. further explored the questions posed by the previous work by specifically analyzing the capillary density detected by NVC in 22 patients with confirmed SARS-CoV-2 infection, who were examined both during hospitalization and 3 months after hospital discharge during the recovery phase. The capillaroscopy results were also correlated with each patient’s laboratory parameters. The considered cohort of patients had bilateral SARS-CoV-2 interstitial pneumonia, and eight patients required noninvasive mechanical ventilation, while six patients presented with thrombotic complications. Capillary density was defined as the number of capillaries per square millimeter of the microscopic field and was counted by hand; in addition, thrombosis, microhemorrhage, and neoangiogenesis of the microcirculation were detected. In the acute phase of the disease, thrombosis (32%), microhemorrhage (36.36%), and neoangiogenesis (27.27%) were detected, which disappeared at the three-month follow-up evaluation. In addition, at the second evaluation, a reduction in capillary density with statistical significance was detected. The basal capillary density was also found to be inversely correlated with the maximal C-reactive protein (CRP) and was directly correlated with the lymphocyte count, but no association with the D-dimer levels was found. This is the first study to re-evaluate patients after hospital discharge by comparing the capillaroscopic pictures of the same subject during and after the acute phase, finding that the microcirculatory changes that occur during SARS-CoV-2 infection are reversible [30].

Çakmak et al. analyzed capillaroscopic changes in 31 children with SARS-CoV-2 infection compared with 58 healthy subjects matched for age and sex. The 31 patients in the COVID-19 group were considered to have a positive swab for SARS-CoV-2 or chest imaging that suggested or demonstrated an increased titer of anti-SARS-CoV-2 antibodies in the blood; of these patients, 6 subjects had aspects of multisystem inflammatory syndrome in children, which is defined as an individual under 21 years of age presenting with fever, laboratory evidence of inflammation, and evidence of clinically severe disease requiring hospitalization, with multisystem (≥2) organ involvement (cardiac, renal, respiratory, hematologic gastrointestinal, dermatologic, or neurologic) without a plausible alternative diagnosis, who is positive for current or recent SARS-CoV-2 infection by real-time polymerase chain reaction (RT-PCR), serology, or antigen testing or who had exposure to COVID-19 in the 4 weeks before the onset of symptoms [31]. The parameters assessed by NVC were similar to those in the previous study, namely, capillary morphology (e.g., tortuosity, crossing, branching, and meandering) and size; defining an enlarged capillary (20 μm–50 μm in diameter) and a giant capillary (diameter ≥50 μm) [22]; the presence of meandering capillaries, which consist of limbs crossed over themselves or with others several times [32]; the presence of pericapillary edema, microhemorrhage, and avascular areas (intercapillary distance greater than 500 μm in the distal capillary row) [33]; and alterations related to neoangiogenesis, such as pathological branching, capillary branching, and the presence of bushy capillaries (multiple small buds originating from the limbs). The scoring system for assessing capillary involvement is different from other studies and involves a score from 0 to 2, defined as 0 = no alterations, 1 = less than 50% of examined capillaries altered, and 2 = more than 50% of examined capillaries altered [26]. In this study, patients with COVID-19 were examined with a median of 73 days post-infection (8–104 days), so considering that the average hospital stay of this group of patients was 5.5 days (4–12), some of them were evaluated during the acute phase, while others were evaluated in the early recovery phase. The group of patients exhibited lower capillary density and length; greater intercapillary distance and tortuosity; and the presence of capillary branching, capillary brushing, brunching, and meandering, with areas of microhemorrhage and neoangiogenesis, all of which presented in a statistically significant manner. 

In addition, capillary changes were more represented in children with higher PCR and D–dimer values [34].

Sulli et al. examined microcirculatory changes in 61 post-COVID patients by comparing the features examined with a healthy control population of 30 patients and 31 subjects with primary Raynaud’s phenomenon. The patients in the post-COVID arm were considered after a mean of 126 ± 53 days from the onset of symptoms and were divided into two subgroups according to the severity of the infection: 34 patients with mild-to-moderate disease, requiring at most low-flow oxygen therapy, and 27 patients with severe infection, requiring invasive or non-invasive mechanical ventilation. Patients with primary Raynaud phenomenon were included according to the LeRoy and Medsger criteria [35], and, in the healthy group, no patients with connective tissue disease or other pathological conditions and therapy possibly influencing the microcirculation were considered.

The NVC parameters considered in this study were 1. dilated capillaries (a capillary diameter between 20 and 50 μm), 2. giant capillaries (homogeneously enlarged loops with a diameter greater than 50 μm), 3. microhemorrhages (dark mass due to hemosiderin deposition), 4. capillary branching (branched or brushed capillaries, a directive sign of neoangiogenesis), and capillary density (fewer than 7 capillaries). Each alteration was scored using the same semi-quantitative assessment as Natallelo et al., with a score ranging from 0 to 3 based on increasing capillary bed involvement [21,22]. Moreover, in this study, the main finding concerns capillary density, which was lower in the post-COVID patient group than in the two control groups. Another interesting finding from this study was that capillary density was less impaired in patients with severe COVID-19 treated with antivirals and IL-6 receptor antagonists during the acute phase of infection, although no statistically significant difference was found, only a positive trend in the reduction in capillary loss. In addition, in the group of patients with previous SARS-CoV-2 infection, capillary branching and dilatation were found, as in previous studies, but none of these features showed true statistical significance compared with the other patient populations considered by Sulli et al. [36]. Table 3 summarizes the statistically significant abnormalities found in adults by NVC in the different studies that were considered.

## 3. NVC vs. Other Methods to Evaluate Microcirculation

The first study to examine microcirculatory alterations in patients with COVID-19 was conducted in Italy by Damiani et al. in April 2020, during the initial phase of the pandemic [37]. The analysis was conducted on the sublingual circulation using incident darkfield video microscopy. The parameters taken into consideration were total vessel density, perfused vessel density, and blood flow quality, in 12 patients admitted to the ICU due to severe SARS-CoV-2 pneumonia. This was the first study to confirm a link between in vivo microcirculation impairment and SARS-CoV-2 infection: the results obtained were not compared with a healthy control; however, by comparing the results with the general population of critically ill ICU patients considered in a previous study conducted by the same authors, a lower density of perfused vessels was found [38]. At the time this study was conducted, the mechanisms underlying SARS-CoV-2 infection were not yet fully understood. The assessment of microcirculation upon admission to the ICU was already shown to be an independent predictor of mortality by Scorcella et al. in the MicroDAIMON study before the pandemic period, but daily monitoring of the sublingual microcirculation did not appear to provide additional prognostic information [38]. Subsequently, Carsetti et al. examined the sublingual circulation in nine patients admitted to the ICU for severe SARS-CoV-2 pneumonia, who underwent extracorporeal venous membrane oxygenation (VV-ECMO) for a lack of response to mechanical ventilation. The alterations in capillary density and microvascular flow parameters were substantially comparable with the results of the patients with severe SARS-CoV-2 pneumonia admitted to the ICU but not subjected to VV-ECMO found by Damiani et al. [37,39]. Furthermore, Carsetti et al. found a relationship between D-dimer values and changes in capillary density, a finding also found by Karahan et al., in patients with severe COVID-19 admitted to intensive care, who were compared with a healthy control group [29].

Simultaneously, Espírito Santo et al. conducted, in 2020, an assessment of microcirculation in patients with severe SARS-CoV-2 infection [40]. Thirteen patients with confirmed severe SARS-CoV-2 disease and on mechanical ventilation were taken into consideration, and the study of the microcirculation was performed using a video capillaroscope placed over the tongue. This study was conducted in the early stages of the pandemic and focused on the search for microthrombi within the microvasculature, which was already found in autopsies on patients with COVID-19 [41]. In particular, evidence of microvascular thrombosis was found in 85% of patients (*n* = 11), a fact also confirmed in studies utilizing NVC by Natalello et al. and Rosei et al. [21,30]. Espirito Santo et al. confirmed the evidence that emerged during the first period of the pandemic, specifically that the systemic involvement of SARS-CoV-2 infection is due to endothelial involvement [7,8,40,41,42,43,44,45,46,47,48,49], so an evaluation of the microcirculation can play a role in patient evaluation. However, the main limitations of this study lie in the absence of a remote evaluation of the patients and in the lack of a control group.

An analysis of a larger patient population, which considered 27 patients with ARDS secondary to SARS-CoV-2 infection admitted to the ICU, intubated and mechanically ventilated, was performed by Kanoore Edul et al. A microcirculation assessment was performed on the sublingual circulation by manual video microscopy. The considered variables were total vascular density, perfused vascular density, proportion of vessels perfused, microvascular flow index, red blood cell velocity, and heterogeneity flow index. This is the only study that found an increase in total vascular density, but again a healthy reference population was not used [42].

Studies conducted by NVC generally looked at larger populations and considered control groups. Some findings from the sublingual assessment were confirmed by more recent data obtained by NVC, as shown above. A study of the microcirculation in the acute phase of the disease, in intubated and mechanically ventilated patients, could be facilitated through a study of the nail circulation. There are currently no studies comparing the results of the two different techniques in patients with SARS-CoV-2 infection; however, both appear to be methods that may reflect systemic involvement. Previously, the possible correlation between the assessment of sublingual and nail microcirculation in patients with systemic sclerosis was analyzed, showing a significant correlation between sublingual video microscopy and NVC in terms of sublingual total microvascular density and the microangiopathy evolution score [43].

Finally, Zharkikh et al. evaluated functional changes in the microvasculature using laser Doppler flowmetry devices in a patient with 10-day daily measurements during COVID-19 and a re-evaluation 26 days after recovery. In a second phase of the study, 26 patients with Long COVID symptoms were evaluated, and the results were compared with those of 13 patients who made up the age-matched healthy control group. SARS-CoV-2 infection causes changes in the microcirculatory blood flow regulation mechanisms, which are measurable by evaluating the spectral characteristics of the laser Doppler flowmetry signal [44,45,46]. The functional alterations of the microcirculation in Long COVID patients seem to confirm the theory that the persistent symptoms after the acute phase are due to lasting endothelial alterations [7]. Furthermore, the functional changes in the microcirculation appear to persist even after the acute phase, unlike the morphological changes that were reversible in the study by Agabiti et al. However, in that Italian study by Agabiti et al., it was not highlighted whether the patients examined in the recovery phase presented symptoms attributable to Long COVID [30].

Other methods widely applicated for evaluating microcirculation are optical coherence tomography (OCT) and optical coherence tomography angiography (OCTA), which are non-invasive imaging techniques that allow for the analysis of quantitative and morphological changes in the retina and ocular microcirculation and are routinely used for the diagnosis and follow-up of diabetic retinopathy, glaucoma, and maculopathies [47]. Several studies demonstrated microvascular ocular alterations in patients with SSc through OCT and OCTA, suggesting their possible applications as a clinical biomarker for assessing disease activity and severity [47,49]. Soysal et al. compared OCTA findings in both adults and children during the acute phase of SARS-CoV-2 with respective healthy control groups: in adults, this study found no statistical OCTA differences between the two subgroups, but, in the pediatric branch, lower capillary density was found, confirming the NVC alterations found in children described by Çakmak et al. [31,50].

Sobi et al. conducted a recent study by performing OCTA in 80 patients with severe acute SARS-CoV-2 infections, after a negative reverse transcription polymerase chain reaction for COVID-19, and documented a lower average retinal vessel density than found in the healthy Indian population; this confirms the result emerging from our review [51]. Actually, Özbaş et al. worked on a larger number of patients who recovered from SARS-CoV-2 (*n* = 105) compared with a healthy control group (*n* = 95) and found no statistically significant difference in the vessel density of the superficial and deep retinal capillary plexus; however, in this study, the patients in the COVID-19 groups presented different severities of the disease [52]. Samendra Karkhur et al. clarified this point by analyzing 180 subjects in a case-control study, compared with healthy vs. recovered from SARS-CoV-2 patients, and pointed out a significant lower vessel density in the more severe form of disease; this finding agrees with Sulli and Agabiti’s results on nailfold examination [30,35,53]. These results were confirmed by other studies such as the COVID-OCTA study, which was conducted in Turkey on 80 patients who had recently recovered from SARS-CoV-2 infections and an 80-patient matching age and sex control group, confirming the lower vessel density on OCTA in the first group, suggesting the use of OCT and OCTA to monitor early changes in diseases affecting microvessels [54]. Even if, at present, there are numerous studies and a larger number of data on OCT and OCTA findings in patients both during and, especially, after SARS-CoV-2 infection, it is reasonable to state that NVC represents a more economic and easily performed way to assess the microcirculation, while OCTA presented a higher risk of imaging artifacts, so the results were more difficult to interpret [55].

## 4. Discussion

Several microcirculatory changes visible by NVC were documented, with different characteristics in the various stages of the disease, from the acute phase to the post-COVID period.

In patients with acute SARS-CoV-2 infection, the main changes are an increase in capillary size (i.e., an enlarged capillary), although never the presence of true giant capillaries, together with edema, microhemorrhages, and thromboembolic phenomena [21,29]. The presence of hemosiderin deposits and capillary alterations are in keeping with the pathophysiological alterations of COVID-19 known to us today, in particular endothelium involvement [7,8]. The several alterations found during the acute phase, regardless of the severity of the infection, are reversible and are no longer found in discharged patients or even in the re-evaluation of the same patients at the resolution of the acute phase [29]. In contrast, the alteration most commonly present in the post-infectious phase is a reduction in capillary density [30,36].

Regarding ICU patients with severe SARS-CoV-2 pneumonia requiring intensive treatments, NVC could be a safe method to assess the patients’ outcome. Several studies before Karahan et al. tested and tried to validate microcirculation impairment as a prognostic factor by evaluating sublingual microcirculation with a sidestream dark field camera, though there were conflicting results regarding the modification of capillary density. In contrast to the sublingual microcirculation assessment, NVC is certainly a safer and more applicable method, due to the fact that patients with severe SARS-CoV-2 pneumonia also often require invasive or non-invasive mechanical ventilation, which counteracts the good performance of the sublingual circulation assessment. A study on a larger sample might be useful to understand whether an evaluation in the first few hours of microcirculation impairment by NVC may be a predictive factor for a patient’s prognosis and, thus, adjust the required intensity of care or predict the course of the acute infection and, therefore, help clinicians in their therapeutic choices [29]. Examining a larger sample of patients would make it possible to investigate the presence of a correlation between the microcirculatory alterations visible by NVC and the development of serious complications secondary to SARS-CoV-2 infection (i.e., pulmonary embolism, ARDS, etc.). The reduction in capillary density appears to be an alteration that occurs in the early stages of inflammation, as noted by Sulli et al., and then persists over time [30,36]. The patients evaluated in the studies in the literature so far were followed up at a maximum of three months, so a longer evaluation over time is necessary to better understand the significance of the reduction in capillary density and possibly assess the presence in that subgroup of patients presenting Long COVID. Finally, the studies available to us so far highlighted the possible usefulness of NVC within SARS-CoV-2 disease, as, among other things, a non-invasive and easily performable method. There does not appear to be a specific pattern of microcirculation alterations related to COVID-19, but non-specific alterations are present that may be of significance for patient management. The impact of SARS-CoV-2 disease on the microcirculation should also be studied on the same patient before and after COVID-19; moreover, with the well-defined and effective pharmaceutical protocols now available, a possible area of interest would be to examine the amelioration of these alterations with more aggressive anti-inflammatory therapy protocols.

**Figure 2:**
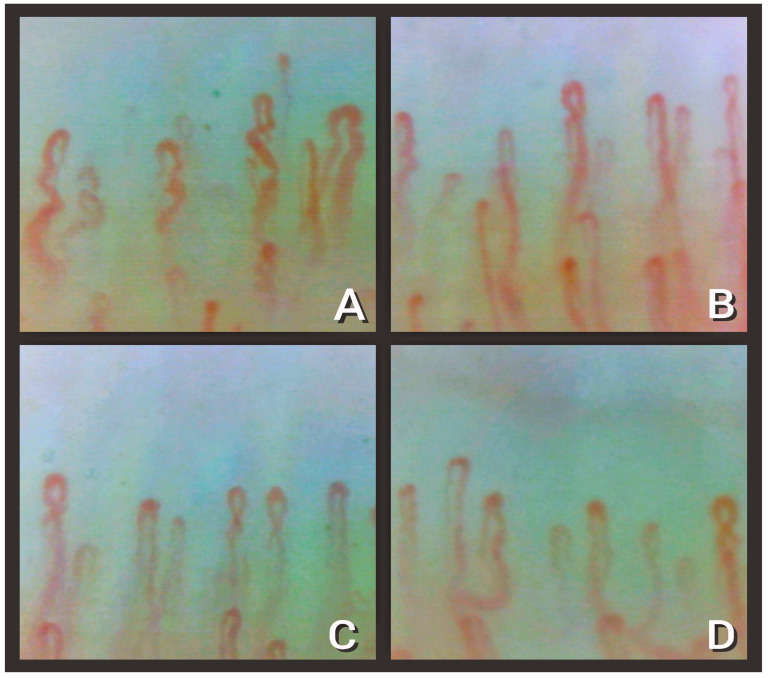
Nailfold video capillaroscopy images (×200) in four COVID-19 survivors (**A**–**D**) with reduced number of capillaries (black bar is one millimeter long) and nonspecific abnormalities (tortuous and crossing capillaries). Images taken by Stefano Tavano, Luca Ruggero, and Lucrezia Mondini.

Figure 2 shows the capillary density reductions found by NVC in patients with SARS-CoV-2 infection.

## 5. Conclusions

NVC is a noninvasive and safe method to assess microcirculation alterations, and its application is not only related to rheumatologic diseases. To date, considering the existing studies, there does not appear to be a pattern for NVC that is specific to SARS-CoV-2 infection, but there are indeed searchable alterations that occur in a statistically significant manner. In the future, it will be interesting to observe how these alterations change over time, focusing on changes in the microcirculation of Long COVID patients, to understand the real weight of each of the visible alterations shown by the NVC regarding the management of the patient, both in terms of the therapeutic choices and treatment in the acute phase and the management of the patient after discharge.

Regarding the acute phase, a more detailed analysis of the data available so far could particularly help with understanding whether there are alterations that may predict or be related to Long COVID forms. Concerning the post-hospitalization phase, NVC could be a non-invasive and easily performable method to monitor the recovery phase and the possible effectiveness of therapeutic and rehabilitation approaches. For this to become clearer, studies on larger samples of patients presenting with Long COVID forms are certainly needed.

## Figures and Tables

**Table 1:** Microvascular alterations seen by nailfold video capillaroscopy and definition adopted for each parameter in the study by Natalello et al., with different involvements in the analyzed subgroups.

Alteration	Definition	Recovered Subgroups (*n* = 28)	Discharged Subgroup (*n* = 54)
*1.Enlarged capillary* [22]	Increased capillary diameter (homogeneous or irregular) > 20 μm and < 50 μm	14.3%	85.2%
2. Giant capillary [22]	Homogeneously enlarged loops (diameter ≥ 50 μm)	0%	0%
*3. Hemosiderin deposit* [23]	Dark mass due to micro-hemorrhage or microthrombosis	46.4%	11.1%
*4. Lower capillary density* [15]	Fewer than 9 capillaries per millimeter	25%	34%
5. Microvascular derangement [22]	Irregular capillary distribution and orientation and heterogeneity of the loops of the same finger	50%	46.3%
6. Capillary ramifications and bizarre morphology [22]	Branching, bushy, or coiled capillaries, often originated from a single normal-sized capillary	28.6%	24.1%
*7. Meandering capillary* [24]	Limbs crossing upon themselves or with each other more than twice	0%	81.4%
*8. Sludge flow* [24]	Markedly slowed or discontinuous flow inside the capillary at the dynamic evaluation at the time of examination	78.6%	40.7%
*9. Pericapillary edema* [25]	Foggy appearance around capillaries due to fluid buildup	100%	40.7%
*10. Subpapillary plexus visibility* [26]	Large and linked arrangement of vessels under the distal row due to enlargement and congestion of venules and capillaries related to persistent opening of arteriovenous anastomoses	3.6%	11.1%
11. Avascular area [27]	Distance > than 500 μm between two adjacent capillary loops from the distal rows	0%	5.6%
*12. Empty dermal papilla* [21]	One or more missing capillaries at the expected place inside dermal papilla, which does not reach the extent to define an avascular area	0%	22.2%

Italics are used the highlight the statistically significant capillary characteristics (*p* value < 0.05).

**Table 2:** Capillaroscopic parameters recorded according to Karahan et al.’s classification.

Alteration	Definition
1. Capillary morphology [28]	Normal, serpentine, or branched
2. Capillary loop diameter [27]	μm diameter at the apical margin of a capillary loop
3. Low capillary density [27]	Number of capillaries in a 1 mm lengthof the distal row of each finger
4. Enlarged capillaries or capillary dilatation [22]	Capillary diameter 20–50 μm
5. Giant capillaries [22]	Capillary diameter > 50 μm
6. Avascular area [27]	Distance between two capillary loops > 500 μm
7. Microaneurysms	Irregular enlargement and circumscribed increase of the capillary loop diameter
8. Microhemorrhages [22]	Hemosiderin deposits with red and/or black images in the distal areas

**Table 3:** Statistically significant capillary alterations found in different studies that were analyzed.

Alteration	Natalello et al. [21]	Karahan et al. [29]	Rosei et al. [30]	Sulli et al. [36]
	54 hospitalized vs. 28 discharged	38 patients in ICU 29 patients healthy	22 patients during and after infection	61 post-COVID (34 mild and 27 severe) vs. 30 healthy patients and 31 patients with RP
Capillary morphology [22]	No statistically significative findings	Serpentine (56% in COVID-19, 20.7% in healthy, *p* < 0.001) Normal (37.5% in COVID-19, 79.5% in healthy, *p* < 0.001)	Not considered	Not considered
Dilatated capillaries [22]	61% in all patients, with 14.3% in acute phase group and 28% in discharged group (*p* < 0.001)	43.8% in COVID-19 vs. 6.9% in healthy control (*p* = 0.001)	Not considered	Present, but not statistically significative
Giant capillaries [22]	Not found (0% in all groups)	Found, but not statistically significative	Not considered	Not found (0% in all groups)
Avascular areas [27]	Not found	Not found	Not considered	Not found
Microaneurysm [29]	Not considered	62.5% in COVID-19 vs. 10.3% in healthy control (*p* < 0.001)	Not considered	Not considered
Hemosiderin deposit [22,23]	19% in all patients, with 46.4% in acute phase group and 11.1% in discharged group (*p* < 0.001)	Considered only as sign of microhemorrhages (listed next)	Separately considered microthrombosis and microhemorrhages	Considered only as sign of microhemorrhages (listed next)
Microthrombosis [23]	7.3% in all patients, with 17.9% in acute phase group and 1.9% in discharged group (*p* = 0.016)	Not considered	Found in 32% patients, not detected after 3 months	Not considered
Microhemorrhages [23]	13% in all patients, with 28.6% in acute phase group and 9.3% in discharged group (*p* = 0.027)	21.9% in COVID-19 vs. 0% in healthy control (*p* < 0.001)	Found in 36% of patients, not detected after 3 months	32.4% in patients with moderate COVID-19; 22.2% in patients with severe COVID-19 64.5% in PRP group 46.7% in control group (*p* univariate 0.005)
Capillary density	Lower than 9/mm in 50% in all patients, with 25% in acute phase group and 63% in discharged group (*p* = 0.002)	6.41 ± 1.21/mm in COVID-19 vs. 8.55 ± 1.12/mm in healthy group	Statistically significant reduction in capillary density after 3 months from the acute infection	8.44 ± 0.75/mm in moderate COVID 8.22 ± 1.15/mm in severe COVID 8.74 ± 0.68/mm in PRP group 9.30 ± 0.53/mm in control group (*p* univariate < 0.001)
